# Thanks to Our Reviewers, *The Plant Genome*, 2021

**DOI:** 10.1002/tpg2.20230

**Published:** 2022-05-23

It is a great pleasure to thank the volunteer reviewers, without whom we could not publish *The Plant Genome*. Despite the demands of their everyday duties, the individuals listed below took time to assist the Editorial Board by reviewing one or more manuscripts in 2021. They demonstrate professionalism in the best sense. For their anonymous, unpaid efforts, all CSSA members owe them thanks. Every effort was made to compile an accurate list. If we have omitted a name, we extend our apologies as well as our thanks.

Ahmad, Suleman, Chinese Academy of Sciences Institute for Nutritional Sciences Shanghai Institutes for Biological Sciences

Akdemir, Deniz

Alok, Anshu, Univ. of Minnesota Twin Cities, USA

Anderson, James, USDA‐ARS, USA

Arya, Lalit, ICAR, India

Ba, Qingsong, Huaibei Normal Univ., China

Babaeian Jelodar, Dr. Nadali, Sari Agricultural Sciences and Natural Resources Univ., Iran (the Islamic Republic of)

Badoni, Saurabh, IRRI, Philippines

Bai, Guihua, USDA‐ARS, USA

Baldrich, Patricia, USA

Barvkar, Vitthal, Univ. of Pune, India

Bayer, Philipp, The Univ. of Western Australia, Australia

Beier, Sebastian, Institute of Plant Genetics and Crop Plant Research (IPK), Germany

Beissinger, Timothy

Belanger, Faith, Rutgers The State Univ. of New Jersey, USA

Bernardo, Rex, Univ. of Minnesota, USA

Bharadwaj, Chellapilla, Indian Agricultural Research Institute, India

Bilyeu, Kristin, USDA‐ARS, USA

Blanco, Antonio, Univ. of Bari, Italy

Bombarely, Aureliano, Universita degli Studi di Milano, Italy

Boyd, Lesley, NIAB, United Kingdom of Great Britain and Northern Ireland

Brasier, Kyle, California State Univ. Chico, USA

Brock, Jordan, Washington Univ. in St Louis, USA

Burgos, Estanislao, Univ. of Zurich Institute of Plant Biology, Switzerland

Burkes, Shelvasha, Univ. of North Carolina at Charlotte, USA

Bustos‐Korts, Daniela, Wageningen Univ., Netherlands

Butardo, Vito, Swinburne Univ. of Technology, Australia

Butt‐Wilmsmeyer, Carrie, Southern Illinois Univ. Edwardsville, USA

Carpentier, Sebastien, KU Leuven, Belgium

Carter, Arron, Washington State Univ., USA

Chamarthi, Siva, Univ. of Arkansas Fayetteville, USA

Chan, Ting‐Fung, The Chinese Univ. of Hong Kong, Hong Kong

Charmet, Gilles, Universite Clermont Auvergne (UCA), USA

Chen, Ming‐Hsuan, USDA‐ARS, USA

Chen, Charles, Oklahoma State Univ., USA

Chen, Shiyu, Univ. of California System, USA

Cheng, Hao, Univ. of California Davis, USA

Cichy, Karen, USDA‐ARS, USA

Clark, Matt, Univ. of Minnesota, USA

Clavijo, Bernardo

Contreras‐Moreira, Bruno, European Molecular Biology Laboratory, United Kingdom of Great Britain and Northern Ireland

Cook, Douglas, Univ. of California Davis, USA

Corak, Keo, Univ. of Wisconsin‐Madison, USA

Cowling, Wallace, The Univ. of Western Australia, Australia

Crain, Jared, Kansas State Univ., USA

Crossa, Jose, CIMMYT, Mexico

Cuevas, Jaime, Universidad de Quintana Roo, Mexico

Cui, Xia, Chinese Academy of Agricultural Sciences Institute of Vegetables and Flowers, China

Daware, Anurag, National Institute of Plant Genome Research, India

de Ronne, Maxime, Université Laval, Canada

DellaPenna, Dean, Michigan State Univ., USA

Derbyshire, Mark, Curtin Univ., Australia

Devanna, BN, Central Rice Research Institute, India

Devkar, Vikas, Texas Tech Univ., USA

Dinglasan, Eric, Cornell Univ. College of Agriculture and Life Sciences, USA

Diouf, Diaga, Université Cheikh Anta Diop de Dakar, Senegal

Dong, Hongxu, Mississippi State Univ., USA

Dong, Zhenying, Univ. of Science and Technology Beijing School of Chemistry and Biological Engineering, China

Dongmei, Dongmei, Henan Agricultural Univ., USA

Dracatos, P. M., Univ. of Sydney, Australia

Dunne, Jeff, North Carolina State Univ., USA

Edme, Serge, USDA‐ARS, USA

Edwards, David, UWA, Australia

Endelman, Jeffrey, Univ. of Wisconsin, USA

Fellers, John, USDA‐ARS, USA

Fernandes, Samuel, Univ. of Illinois at Urbana‐Champaign, USA

Ferreira, Juan Jose, SERIDA, Spain

Fritsche Net, Roberto

Fu, Junjie, Chinese Academy of Agricultural Sciences Institute of Crop Sciences, China

Fu, Xingzheng, Southwest Univ.

Fujimoto, Ryo, Kobe Univ.

Funnell‐Harris, Deanna, USDA ARS, USA

Garland‐Campbell, Kim, USDA ARS, USA

Garnatje, Teresa, CSIC ‐ Institut Botanic de Barcelona (ICUB), USA

Gharanjik, Dr. Shahrokh, Shahrood Univ., Iran (the Islamic Republic of)

Gomez, Francisco, Michigan State Univ., USA

Gorjanc, Gregor, The Roslin Institute and Royal (Dick) School of Veterinary Studies, United Kingdom of Great Britain and Northern Ireland

Gundlach, Heidrun

Gutierrez, Lucia, Univ. of Wisconsin Madison, USA

Haas, Matthew, Univ. of Minnesota Twin Cities, USA

Haberer, Georg, Helmholtz Zentrum Munchen Deutsches Forschungszentrum fur Umwelt und Gesundheit, Germany

Haley, Scott, Colorado State Univ., USA

Harrasi, Ibtisam Al

He, Guohao, Tuskegee Univ., USA

He, Hang, Peking Univ.

He, Yuqing

Hedrich, Rainer, Univ. of Würzburg

Henry, Robert, The Univ. of Queensland ‐ Saint Lucia Campus, Australia

Herath, Venura, Univ. of Peradeniya, Sri Lanka

Hong, Yangbin, CRI‐GDAAS, China

Horner, David S., Università degli Studi di Milano, Italy

Hua, Jinping, China

Hudson, Matthew E., Univ. of Illinois System, USA

Hufnagel, Bárbara, France

Imran, Quari, Umea Univ., Sweden

Jackson, Phillip, CSIRO, Australia

Jackson, Scott A.

Jarquin, Diego, Univ. of Nebraska‐Lincoln, USA

Jayakodi, Murukarthick, IPK‐Gatersleben, Germany

Jin, Biao, Yangzhou Univ., China

Kang, Yichen, The Univ. of Queensland ‐ Saint Lucia Campus, Australia

Kantar, Michael, Univ. of Hawaii at Manoa, USA

Katara, Jawahar, ICAR‐National Rice Research Institute, India

Kitazumi, Ai, Texas Tech Univ., USA

Klein, Robert, USDA‐ARS, USA

Kong, Fanjiang, Guangzhou Univ., China

Kosik, Ondrej, Rothamsted Research, United Kingdom of Great Britain and Northern Ireland

Krause, Margaret, Utah State Univ.

Kumar, Anuj, Univ. of Arkansas Fayetteville, USA

Kuraparthy, Vasu, North Carolina State Univ., USA

Kurihara, Yukio, RIKEN, USA

Lakhssassi, Naoufal, Southern Illinois Univ. System, USA

Lee, JaeSung

Lee, Suk‐Ha, Seoul National Univ., Korea (the Republic of)

Lee, Sungwoo, Chungnam National Univ., Korea (the Republic of)

Legarra, Andres, INRAE, USA

Levinson, Chandler, Univ. of California Davis, USA

Li, Shuai, Qingdao Agricultural Univ., China

Li, Huihui, Institute of Crop Science, China

Li, Man‐Wah, The Chinese Univ. of Hong Kong, Hong Kong

Li, Peirong, Beijing Vegetable Research Center, China

Li, Xin, AgReliant Genetics, USA

Lipka, Alexander, Univ. of Illinois at Urbana‐Champaign, USA

Liu, Shuyu, Texas A&M Univ. System, USA

Liu, Rong, Chinese Academy of Agricultural Sciences Institute of Crop Sciences, China

Liu, Qiao‐Quan, Yangzhou Univ., China

Liu, Wenxin, China Agricultural Univ., China

Loopstra, Carol

Lopes, Marta da Silva Sabino, IRTA Institute of Agrifood Research and Technology, USA

Lorenz, Aaron, USA

Lu, Kun, Southwest Univ., China

Luque, Francisco, Universidad de Jaen, Spain

Ma, Tao, Sichuan Univ., China

Mascher, Martin, IPK Gatersleben, Germany

McCabe, Matthew, King Abdullah Univ. of Science and Technology, Saudi Arabia

McClean, Phillip, North Dakota State Univ., USA

McHale, Leah, Ohio State Univ., USA

Megerssa, Shitaye, Cornell Univ. College of Agriculture and Life Sciences, USA

Melonek, Joanna, ARC Centre of Excellence in Plant Energy Biology, Australia

Michel, Sebastian, Univ. of Life Science Vienna, Austria

Millar, Anthony A., Australian National Univ.

Milner, Matthew, NIAB, United Kingdom of Great Britain and Northern Ireland

Miranda, Carrie, North Dakota State Univ., USA

Monat, Cécile, Syngenta France SAS, France

Monhanty, Bijayalaxmi, National Univ. of Singapore ‐ Kent Ridge Campus, Singapore

Mosa, Kareem, Univ. of Sharjah, United Arab Emirates

Munaiz, Eduardo, Mision Biologica de Galicia, Spain

Munoz, Patricio, Univ. of Florida, USA

Muñoz‐Amatriaín, María, Colorado State Univ., USA

Naegele, Rachel, USDA‐ARS, USA

Najafabadi, Mohsen Yoosefzadeh, Univ. of Guelph Ontario Agricultural College, Canada

Nanda, Satybhrata, Centurion Univ., India

N'Diaye, Amidou, Univ. of Saskatchewan, Canada

Obermeier, Christian, Univ. of Giessen

Ohyanagi, Hajime

Oldroyd, Giles, Sainsbury Laboratory, United Kingdom of Great Britain and Northern Ireland

Ongom, Patrick, IITA, Nigeria

Owens, Gregory

Pabuayon, Isaiah, Texas Tech Univ., USA

Pandey, Manish, ICRISAT, India

Parkin, Isobel, Agriculture and Agri‐Food Canada, Canada

Passianotto, André, BASF Brasil, Brazil

Patil, Gunvant, Texas Tech Univ., USA

Paule, Juraj, Nat Hist Museum Frankfurt, USA

Pauls, Peter, Univ. of Guelph, Canada

Pembleton, Luke, AgriBio, Australia

Pepper, Alan, Texas A&M Univ., USA

Pérez‐Enciso, Miguel, Center for Research in Agricultural Genomics

Perez‐Rodriguez, Paulino, Colegio de Postgraduados, Mexico

Periyannan, Sambasivam, CSIRO Plant Ind, Australia

Porch, Timothy, USDA‐ARS, Puerto Rico

Posadas, Luis, Univ. of Nebraska‐Lincoln, USA

Pourkheirandish, Mohammad, Univ. of Melbourne Faculty of Veterinary and Agricultural Sciences, Australia

Powell, Owen, Queensland Alliance for Agriculture and Food Innovation, Australia

Pradhan, Sharat, ICAR‐National Rice Research Institute, India

Prasad, Manoj, National Institute of Plant Genome Research, India

Pucker, Boas, Univ. of Cambridge, United Kingdom of Great Britain and Northern Ireland

Qiu, Xianjin, Yangtze Univ, China

Rabanus‐Wallace, Mark Timothy

Rahman, Sadequr, Monash Univ. Malaysia, Malaysia

Ramirez‐Gonzalez, Ricardo, John Innes Centre, United Kingdom of Great Britain and Northern Ireland

Reid, Robert, USA

Reif, Jochen, Department of Leibniz Institute of Plant Genetics and Crop Plant Research (IPK), Germany

Resende Jr., Marcio, Univ. of Florida, USA

Riday, Heathcliffe

Rife, Trevor, Kansas State Univ., USA

Robbins, Kelly, Cornell Univ. College of Agriculture and Life Sciences, USA

Robins, Joseph, USDA‐ARS Forage and Range, USA

Roose, Mikeal L., Univ. of California Riverside Department of Botany & Plant Sciences, USA

Rouse, Matthew N., USDA‐ARS, USA

Ru, Sushan, Auburn Univ. System, USA

Sabot, Francois, IRD France Sud, France

Salas Fernandez, Maria, Iowa State Univ., USA

Sallam, Ahmad, Univ. of Minnesota, USA

Sanchez‐Martin, Javier

Sandhu, Devinder, USDA‐ARS, USA

Sandhu, Karansher, Washington State Univ., USA

Santantonio, Nicholas, Virginia Tech, USA

Sapkota, Surya, Cornell Univ., USA

Sapkota, Suraj, USDA‐ARS SEA, USA

Schlueter, Jessica, UNC‐Charlotte, USA

Schnable, James, Univ. of Nebraska, Lincoln, USA

Seraj, Zeba, Univ. of Dhaka, Bangladesh

Serumaga, Julius, National Agricultural Research Organisation, Uganda

Sharbel, Tim, Univ. of Saskatchewan,

Shen, Qingxi J., Univ. of Nevada Las Vegas, USA

Shenton, Matthew, NARO, Japan

Shi, Ainong, Univ. of Arkansas, USA

Shiu, Shin‐Han, Michigan State Univ., USA

Shivaraj, SM, National Chemical Laboratory CSIR, India

Sims, Jason, Univ. of Vienna Faculty of Life Sciences, Austria

Singh, Rakesh, National Bureau of Plant Genetic Resources, India

Singh, D., The Univ. of Sydney Plant Breeding Institute, Australia

Singh, Kashmir, Panjab Univ.

Singh, Rakesh, International Center for Biosaline Agriculture, United Arab Emirates

Singh, Vikas, IRRI, India

Smith, Kevin P., Univ. of Minnesota, USA

Smykal, Petr, Palacky Univ. in Olomouc, Czech Republic

Snowdon, Rod, Justus Liebig Univ., Giessen, Germany

Sonah, Humira, Laval Univ., Canada

Song, Qijian

Song, Qijian, USDA‐ARS, USA

Song, Shun, Chinese Academy of Tropical Agricultural Sciences, China

Spannagl, Manuel, Helmholtz Center Munich, Germany

Stanislaus, Antony Ceasar, Universite de Liege Departement des Sciences de la Vie, Belgium

Stein, Nils, Leibniz Institute of Plant Genetics and Crop Plant Research (IPK), Germany

Sukumaran, Sivakumar, CIMMYT INT, Mexico

Tan, Ruijuan

Tao, Yongfu, Univ. of Queensland, Australia

Temsch, Eva M., Univ. of Vienna, USA

Tetreault, Hannah, USDA, USA

Tibbs‐Cortes, Laura, Iowa State Univ., USA

Toth, Jacob, Cornell Univ., USA

Tracy, William, Univ. of Wisconsin‐Madison, USA

Tuskan, Gerald, US Department of Energy Center for Bioenergy Innovation, USA

Uauy, Cristobal

Upadhyay, Santosh, National Agri‐Food Biotechnology Institute, India

Van Sanford, David, Univ. of Kentucky, USA

Vandepoele, Klaas, Univ Ghent, Belgium

Vanous, Adam, USDA‐ARS, USA

Varotto, Serena, Univ. of Padova, Italy

Von Wettberg, Eric B.

Wang, Guifeng, Henan Agricultural Univ., China

Wang, Jiankang

Wang, Mingle, Huazhong Agricultural Univ., China

Wang, Xingjun, Shandong Academy of Agricultural Sciences

Wang, Jinyu, Iowa State Univ., USA

Ward, Brian P., Virginia Tech, USA

Ward, Brian, USDA‐ARS, USA

Warzecha, Tomasz, Univ. of Agriculture in Krakow, Poland

Whitaker, Vance, Univ. of Florida IFAS, USA

Worthington, Margaret, Univ. of Arkansas, USA

Xia, Rui

Xu, Pei

Xu, Shutu, Northwest A&F Univ., China

Xu, Xiangyang, USDA‐ARS, USA

Yan, Long, Hebei Academy of Agricultural and Forestry Sciences, China

Yang, Sihai, Nanjing Univ., China

Yang, Yan

Yu, Jianming, Iowa State Univ., USA

Yuan, Youlu, Chinese Acad. Agr. Sci., China

Zhang, Zhiwu, Washington State Univ., USA

Zhang, Chaozhong, Univ. of California System, USA

Zhang, Dajian, Shandong Agricultural Univ., China

Zhang, Dapeng, USDA‐ARS Beltsville Agricultural Research Center, USA

Zhang, Lei, Purdue Univ.

Zhang, Qiong

Zhang, Xuecai, International Maize and Wheat Improvement Center (CIMMYT), Mexico

Zhang, Jiaoping, Nanjing Agricultural Univ., China

Zhao, Jiantao, Cornell Univ., USA

Zhao, Tuanjie

Zhao, Yusheng, Leibniz Institute of Plant Genetics and Crop Plant Research (IPK), Germany

Zhuang, Weijian, Fujian Agriculture and Forestry Univ., China

Zurn, Jason, Kansas State Univ., USA

